# Tribological Properties Laser-Cladded Spherical WB-Reinforced Co-Based Coatings under Low-Temperature Friction

**DOI:** 10.3390/ma16196444

**Published:** 2023-09-27

**Authors:** Li Fan, Haiyan Chen, Guangkuo Zhu, Qizheng Cao, Lihua Dong

**Affiliations:** 1 College of Mechanical and Electronic Engineering, Shanghai Jian Qiao University, Shanghai 201306, China; 2 College of Ocean Science and Engineering, Shanghai Maritime University, Shanghai 201306, China; 202230410116@stu.shmtu.edu.cn (G.Z.); lhdong@shmtu.edu.cn (L.D.); 3 College of Materials Science and Engineering, Shanghai University, Shanghai 200072, China; caoqizheng2020@163.com; 4 State Key Laboratory of Advanced Special Steel, Shanghai University, Shanghai 200072, China

**Keywords:** low-temperature friction, tribological behavior, Co-based coating, spherical tungsten boride (WB)

## Abstract

Three groups of spherical WB-reinforced Co-based coatings (Co coating, Co + 15%WB coating, Co + 45%WB coating) were fabricated by laser-cladded technology. The microstructure and constituent phase of spherical WB-reinforced Co-based coatings were examined through scanning electron microscopy (SEM) with energy dispersive spectrometry (EDS) and X-ray diffraction (XRD). The low-temperature tribological properties were analyzed by coefficient of friction, 2D and 3D profiles across the wear track, and wear surface morphology, respectively. The results showed that the phases in the WB-reinforced Co-based coatings are mainly γ-Co, carbides Cr_23_C_6_ and Cr_7_C_3_, WB, and WO_3_. Under dry sliding friction at −20 °C, the more spherical WB, the lower the friction coefficient. The wear rate of Co + 45%WB coating was as low as 3.567 × 10^−4^ mm^3^/N·m^−1^, indicating the outstanding wear resistance. Abrasive wear was observed on the rough surface of the WB-added coatings. Compared with dry sliding, due to the plastic deformation of micro-convexes and lubrication function in the 3.5 wt.% NaCl solution, the wear tracks on the surfaces of the three tested coatings were shallower, exhibiting distinct elongated plough grooves.

## 1. Introduction

With global warming and the increase in human activities in the Arctic region, Arctic-related research has become increasingly prominent [1,2]. Higher requirements have been put forward for polar ships and engineering materials that can withstand harsh conditions and low temperatures [3,4]. Serious wear and corrosion, or even erosive-corrosive wear, are the most important failure of polar ship hull steels. Surface protective coatings are very promising alternatives to increase the wear and corrosion resistance of the substrate steel.

Lots of surface protection techniques such as plasma transferred arc (PTA), thermal spray, and laser-clad have been widely investigated. Compared with other surface treatment technology, laser-clad method has several advantages, such as low dilution [5], high energy density, superior mechanical properties [6], lower metallurgical defects, and a strong metallurgical bonding with the substrate [7,8]. It is considered as one of the most important and promising candidate for surface protective coatings.

Ceramic-metallic coatings are generally known for their superior performances under aggressive environments, such as high wear and corrosion resistance [9,10]. The metallic binder provides the coatings with excellent ductility and toughness, while the reinforced ceramic particle provides the coatings with high hardness and wear resistance [11,12,13]. These features make ceramic-metallic coatings attractive in surface protective application. In the ceramic-metallic coatings, the bonding metallic matrix can be Co-based, Ni-based or Fe-based alloys. The reinforced phases mainly consist of carbide, boride, nitride, oxide, and other ceramic particles.

Extensive studies have focused on boride ceramic materials with ultra-high hardness [14,15,16]. Ultra-hard materials represented by transition metal borides (with a hardness value above 40 GPa) exhibit remarkable properties such as high hardness, excellent wear resistance, and outstanding chemical stability. Boron, as a unique non-metallic element, can form various hybridized covalent bonding forms (sp, sp2, sp3) and possesses both electron accepting and electron donating abilities, resulting in the formation of diverse compounds with transition metals [17].

Different tungsten boride compounds (WB, WB_2_ WB_3_, and WB_4_) exhibit distinct mechanical properties. Among them, WB has great potential value in engineering applications. Numerous studies have been focused on tungsten boride strengthened Co-based coatings. Wang et al. [17] prepared a WC-based coating using HVOF with the binder of ultra-hard WCoB compound. The results showed that compared to WC-Co coatings, the WC-WCoB coating simultaneously increased hardness and fracture toughness, significantly reducing the wear rate. The improved frictional performance of the WC-WCoB coating was attributed to reduced plastic deformation, which inhibited micro-wear. Additionally, the high hardness of WCoB and its good fusion provided better resistance to wear compared to traditional coatings. Yan et al. [18] prepared a high-temperature oxidation and wear-resistant coating by thermal spraying WB-doped WC-Co powder. The addition of WB led to the in situ formation of WCoB. Thermal analysis showed that the oxidation resistance of WCoB at high temperatures was significantly higher than that of WC and Co. Therefore, the oxidation of the WC-WCoB coating was mainly initiated by WC, and after high-temperature wear tests, most of the WCoB remained in the coating. The wear rate of the coating was significantly reduced by 90% and 77% at room temperature and high temperature, respectively. Munteanu et al. [19] deposited thin wear-resistant coatings of 20 (Ni17.5Cr4Fe4Si4B 0.5C), 80 (WC12Co), and 88WC12Co metal powders through atmospheric plasma spraying (APS). Sun et al. [20] synthesized WCoB/WNiB phases in situ by the reaction of WB with Co/Ni, and a series of novel WB-WCoB coatings were prepared by controlling the content of the metal phases. The oxidation resistance of WB-WCoB coatings was significantly better than that of WC-Co coatings, and with an increase in WCoB content, the wear resistance of WB-WCoB coatings under high-temperature conditions improved. Due to the lower electrode potential of WCoB compared to WB, WB-based coatings with lower WCoB content exhibited better corrosion resistance.

All of the above literatures are mainly focused on the general properties of WB-reinforced coatings and their wear resistance under room or high temperatures. However, few studies have been conducted on wear resistance of WB-reinforced coatings under low temperature. Thus, the outcomes on low-temperature tribological properties, laser-cladded, spherical WB-reinforced, Co-based coatings being a novelty.

In this study, laser-cladding technology was employed to prepare three groups of spherical WB-reinforced Co-based coatings. The influence of different WB contents on microstructural strengthening mechanism, as well as the microhardness evolution and low-temperature tribological behaviors of the coatings were analyzed.

## 2. Materials and Methods

### 2.1. Materials

**Figure 1:**
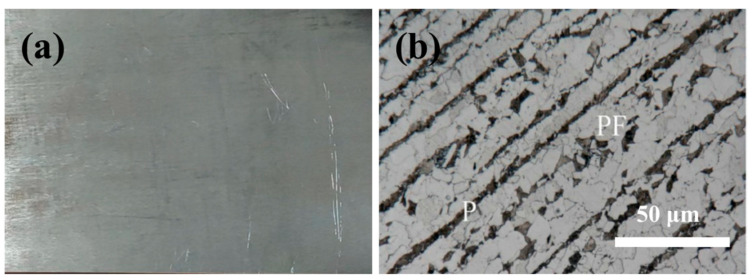
Substrate steel morphology: (**a**) Macroscopic morphology; (**b**) metallographic structure.

The substrate material used in the experiment was EH40 high-strength low-temperature steel, obtained from China Baowu Steel Group Corporation. The chemical compositions (wt.%) of EH40 steel were 0.16 C, 0.15 Si, 0.90 Mn, 0.10 V, 0.20 Cr, 0.02 Nb, 0.020 Al, 0.08 Mo, 0.35 Cu, 0.40 Ni, 0.02 Ti, and Fe balance. The EH40 steel was in the normalized condition and consisted mainly of polygonal ferrite (PF) and pearlite (P) structures. Prior to the cladding process, the surface of the EH40 steel was ground using 80# sandpaper to remove rust layers. Macroscopic morphology and metallographic structure of EH40 substrate steel are shown in Figure 1.

**Figure 2:**
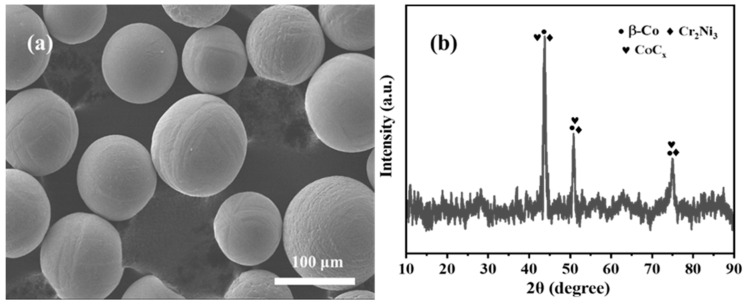
Co-based alloy powder: (**a**) Micromorphology; (**b**) XRD pattern.

Co-based alloy powder (Höganäs, Shanghai, China) with the elemental composition (wt.%) of 1.4 C, 7.7 W, 0.2 Ni, 0.2 Fe, 27.6 Cr, 1 Si, and Co balance was used as the binder phase for the composite coatings. The micromorphology of the Co-based powder was observed using JEOL JSM-7500F scanning electron microscope (SEM, JEOL, Tokyo, Japan), and the powder phase compositions were examined by PANalytical X’Pert PRO X-ray diffractometer (XRD, PANalytical B.V., Almelo, The Netherlands) with a Cu-Kα radiation over a 2θ range of 20°–80°. The results of SEM and XRD are shown in Figure 2. Figure 2a shows that the powder has uniform particle size and good sphericity, ensuring smooth flow during the cladding process.

### 2.2. Spheroidization of WB

**Figure 3:**
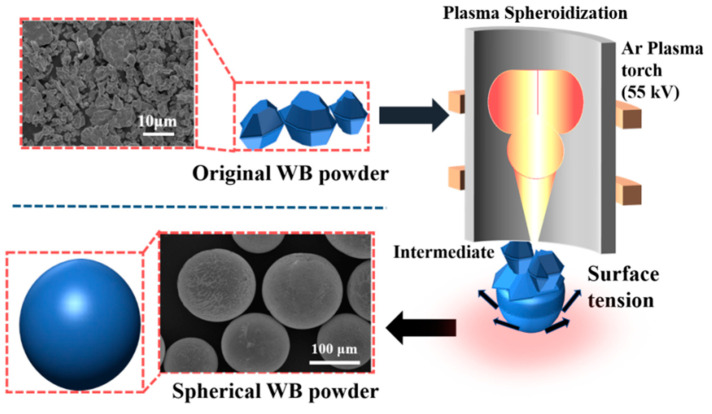
Schematic diagram of spheroidizing WB process.

The original WB particles (produced by Luoyang JinLu Hard Alloy Co., Ltd., Luoyang, China) used in the experiment were in irregular shape. When used in surfacing coating, irregular powders are easy to cause stress concentration and cracks under load, thus leading to performance defects of the surfacing coating [21]. The spheroidization of WB particles was carried out on the RF induction plasma equipment manufactured by Tekna (Canada). The schematic diagram of the spheroidization process is shown in Figure 3. Argon gas was used as the carrier gas, sheath gas, dispersing gas, and central gas during the spheroidization process. The detailed parameters of the spheroidization process are presented in Table 1.

**Figure 4:**
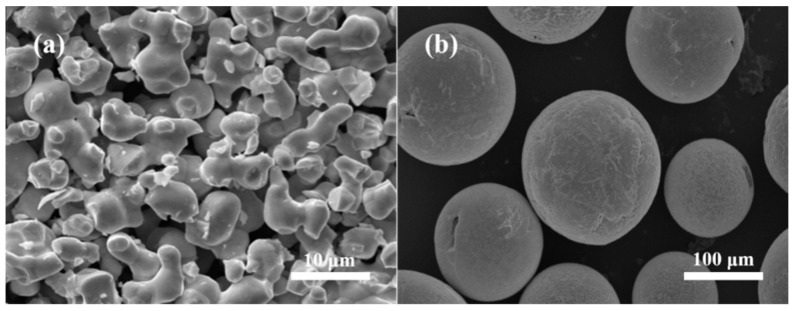
The SEM morphology of WB powder: (**a**) before spheroidization; (**b**) after spheroidization.

**Figure 5:**
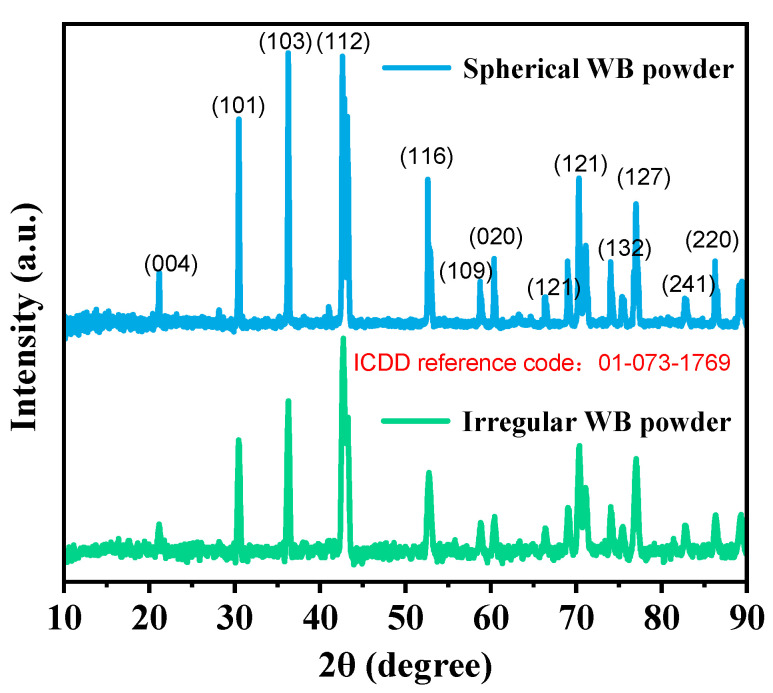
XRD patterns of WB powder before and after spheroidization.

The scanning electron microscopy (SEM) morphologies of WB powder before and after spheroidization are shown in Figure 4. The spheroidization rate reached 99%, and the particle size ranged from 45 to 200 μm. Figure 5 presents the XRD patterns of WB powder before and after spheroidization. The principal phase compositions were identical, but the peaks in the XRD pattern of the spheroidized powder between 20° and 45° were sharper, indicating an improvement in crystallinity after spheroidization.

### 2.3. Sample Preparation and Characterization

**Figure 6:**
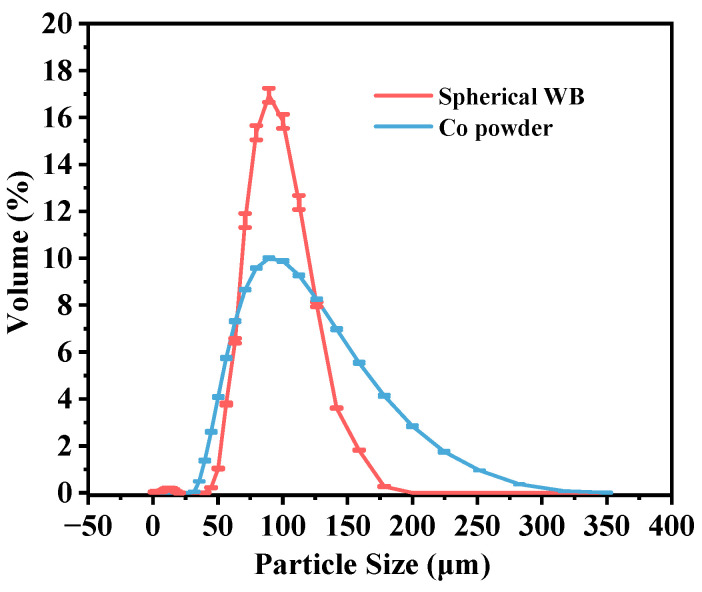
Particle size distribution of Co-based and spherical WB powders.

The Co-based alloy powder and the spherical WB powder were mixed in the predetermined ratio as shown in Table 2. The particle size distribution of the two powders is illustrated in Figure 6. The particle size of the Co-based powder ranged from 25 to 325 μm, while the spherical WB powder had a particle size ranging from 45 to 200 μm. Both powders exhibited a Gaussian distribution. The mixed powders were placed in a QM-3SP4 planetary ball mill (Nanjing Nanda Instrument Co., Ltd., Nanjing, China) for mechanical mixing to 1 h to ensure a homogeneous mixture of the two powders.

**Figure 7:**
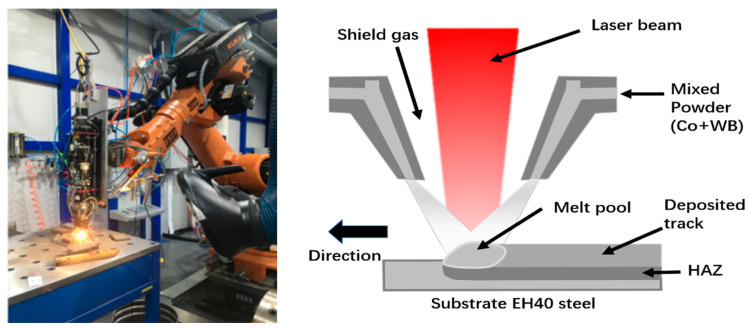
Schematic diagram of laser cladded equipment and process.

The laser cladding experiments were conducted using a high-power fiber laser (YLS-6000-S2T, IPG, New York, NY, USA) with a wavelength of 1075 ± 5 nm and a maximum output power of 5.5 kW. The laser beam was rectangular with size of 10 mm × 2 mm, and the laser focal length was 150 mm. Single-pass laser with multi-track overlapping was used, with argon (Ar) as the shielding gas. The flow rate of shielding Ar was 10 L/min. The process parameters for laser cladding in this experiment are listed in Table 3. The laser-cladding equipment and the schematic of the cladding process are shown in Figure 7. The samples were then cut by wire electrical discharge machining (EDM) into specimens with dimensions of 10 mm × 10 mm × 5 mm for microscopic morphology and phase analysis, hardness testing, and low temperature friction-wear test.

Prior to microstructure observation, the coating was progressively polished using sandpaper from 80# to 2000#, followed by polishing. Then, the cross-section and surface of the specimens were etched with aqua regia (HCl:HNO_3_ = 3:1) for metallography observation. The microstructure of the laser cladded coating was observed using a JEOL JSM-7500F scanning electron microscope (SEM, JEOL, Tokyo, Japan) equipped with an energy-dispersive spectrometer (EDS).

The phase analysis of the Co-based WB coating was performed using a PANalytical X’Pert PRO X-ray diffractometer (XRD, PANalytical B.V., Almelo, The Netherlands). The XRD instrument utilized Cu-Kα radiation (λ = 0.154060 nm) with an operating voltage of 40 kV and a current of 40 mA. The scanning range was set from 10° to 90° with a scan speed of 0.3145°/s and a step size of 0.02°. The data analysis was conducted using the High Score Plus software (Version 5.1) to determine the phase composition of the Co-based WB coating.

### 2.4. Low Temperature Friction and Hardness Tests

The microhardness distribution of the Co-based WB coating was measured using an HXD-20007M/LCD Vickers hardness tester (Shanghai TaiMing Optical Instrument, Shanghai, China). A load of 1 kg was applied for 15 s, and the diamond indenter measured one point per 100 μm on the cross-section of the sample. Three parallel experiments were conducted to reduce errors, and the hardness tests were performed at room temperature.

The Co-based WB coating was subjected to reciprocating linear friction and wear tests under preloaded normal load using a BRUKER UMT TriboLab tribometer (BRUKER, Luken, Germany). The WC ball with a diameter of 8 mm served as the counterpart. The experiments were conducted with a loading force of 50 N, a frequency of 2 Hz, an amplitude of 5 mm, a test duration of 60 min, and a total sliding distance of 72 m. The tests were performed at a low temperature of −20 °C under dry sliding conditions and wet sliding conditions with a 3.5 wt.% NaCl solution as the lubricant. Three repeated-wear tests were undertaken for each sample to ensure the reliability and reproducibility of the results. The coefficients of friction (COF) value was continuously recorded by the TriboLab software, and the average COF values were given as the steady friction coefficient. After the tribological tests, the worn surfaces were then cleaned with ethanol and dried with an electrical drier. The surface morphology and depth of the wear tracks of the coating were observed using a BRUKER ContourGT 3D optical profilometer (BRUKER, Luken, Germany).

## 3. Results and Discussion

### 3.1. Microstructure and Constituent Phase of the Laser Cladded Coatings

**Figure 8:**
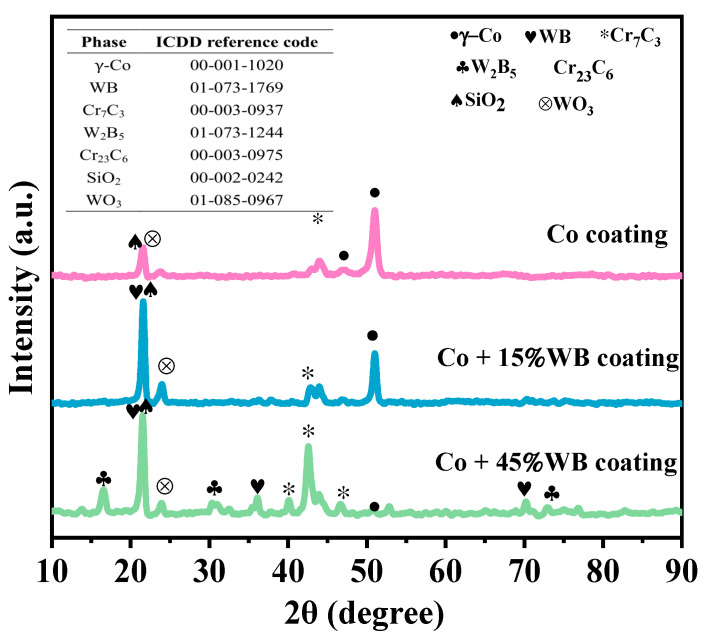
XRD diffraction patterns of cladding layers with different WB content.

Figure 8 shows the XRD spectra of Co-based WB coatings. The γ-Co (FCC) solid solution, Cr_7_C_3_, WO_3_, and SiO_2_ were detected in all coatings. Due to the addition of Cr, C, and Si elements in the Co-based powder, Si element oxidized to form SiO_2_ during the coating process. Si element exhibits self-melting behavior and its addition refines the alloy grains, promoting the transformation from equiaxed to dendritic structure [22]. Cr_7_C_3_ is one kind of carbide formed by the chemical reaction of Cr and C elements under laser energy. In addition to the Cr_7_C_3_ phase, intermetallic compounds such as Cr_23_C_6_ carbide was also detected, which play a role in dispersion strengthening of the coating. The Cr_23_C_6_ intermetallic compound has a complex cubic structure, which is relatively stable and requires high energy to form a new phase composed of atoms. It can also facilitate the long-range diffusion of other atoms. The intensity of the γ-Co diffraction peak significantly decreases with decreasing Co content. WB with a high melting point is not easily melted by laser energy, as seen in the spectrum where a significant amount of WB particles remain in the coating. It has been reported that WC can decarburize to form W_2_C and C under thermal reaction conditions [23]. As the content of WB gradually increases, a portion of WB will also decompose into W_2_B_5_. The W element reacts with oxygen to form WO_3_ oxide. A broadening diffuse scattering peak appears in the Co and Co + 15%WB coatings between 40° and 45°, indicating the presence of an amorphous structure.

**Figure 9:**
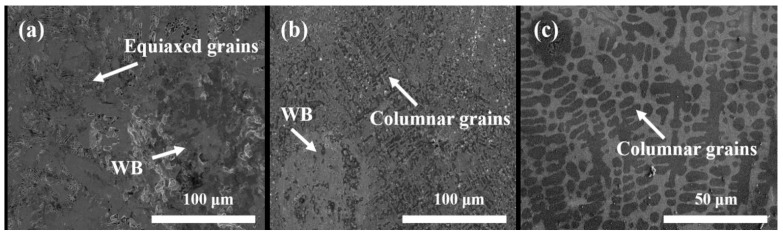
SEM of laser-cladded coatings: (**a**) Co + 15%WB; (**b**) Co + 45%WB; (**c**) Co.

Figure 9 shows the surface microstructure of Co-based WB coatings. In Figure 9a,b, some voids and defects are observed within and around the WB particles. According to previous studies, during the rapid cooling process of the molten pool, some gases are trapped by WB and cannot quickly escape due to solidification at high temperatures [24]. Large areas of equiaxed grains are distributed around the spherical WB particles, and in the Co + 15%WB coating, predominantly fine equiaxed dendritic structures are present. For the Co + 45%WB coating, it can be observed that when a significant amount of large-sized WB particles is present, some columnar grain zones extend outward from the WB particles. This is attributed to the Particle Stimulated Nucleation (PSN) phenomenon [25] where a larger amount of WB particles can induce nucleation and accelerate recrystallization. Figure 9c reveals that the Co coating exhibits predominantly columnar dendritic microstructure, which is uniform and well-defined, with essentially no amorphous phase [26]. Without the reinforced spherical WB particles, the cobalt-based alloy powder absorbs more laser energy, resulting in a smaller temperature gradient during cooling, providing sufficient time for crystal growth and the formation of coarser columnar dendrites.

**Figure 10:**
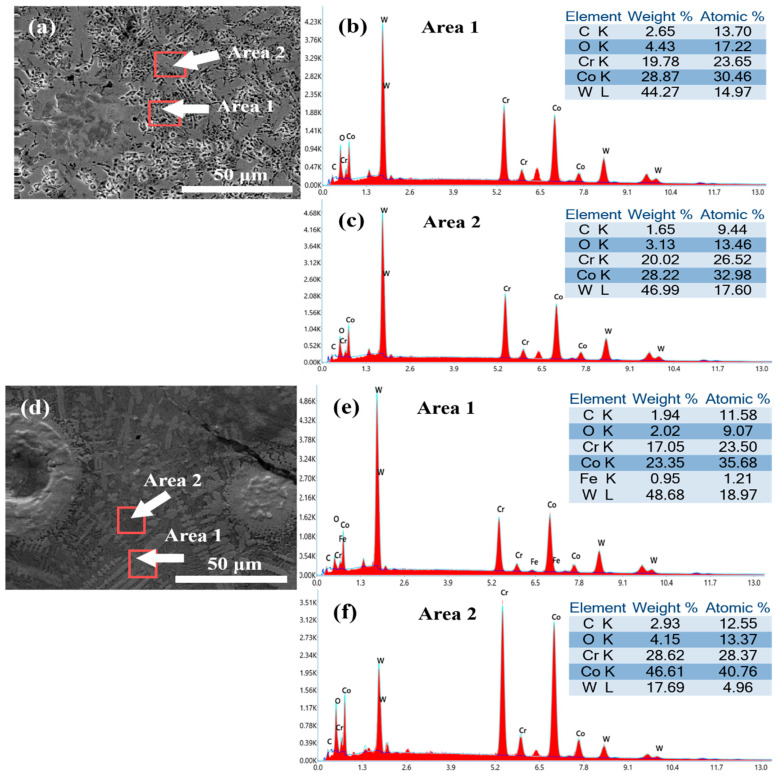

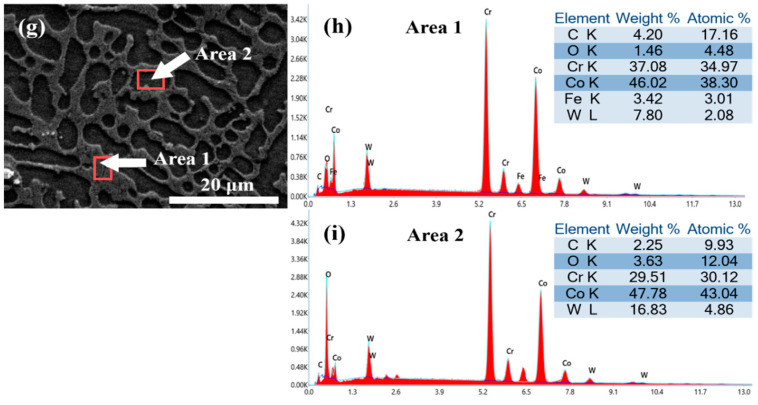
Surface EDS spectrum of laser-cladded coatings: (**a**–**c**) Co + 15%WB; (**d**–**f**) Co + 45%WB; (**g**–**i**) Co.

To gain a better understanding of the microstructural element distribution in the coating, EDS scanning analysis was conducted on the gray-black eutectic structure and white dendritic structure of the coating, as shown in Figure 10. The elemental distribution in the Co + 15%WB region is depicted in Figure 10a–c, revealing significant enrichment of Co and W elements around the spherical WB particles. The mass fraction of W element reached approximately 45%. Combined with the XRD results, numerous WO_3_ and SiO_2_ phases were distributed around the spherical WB particles. Additionally, some carbide reinforcement phases were surrounded by the γ-Co solid solution. Based on the atomic ratio of elements, the interdendritic structure mainly consisted of Cr_7_C_3_, while the white dendritic structure primarily comprised Cr_23_C_6._ The element content in the corresponding region of the Co + 45%WB coating is shown in Figure 10d–f. Combining the XRD results with the element content in the corresponding region, it can be determined that Area 1 was mainly composed of columnar grains with abundant elements such as Co, Cr, and W. The main components in this area were Cr_7_C_3_ carbide and WO_3_. Area 2 represents eutectic regions between columnar grains, where γ-Co dominated the composition, and Cr_7_C_3_ carbide and WO_3_ contributed to dispersion strengthening within the γ-Co matrix. With the presence of a larger amount of WB, the slower cooling rate allowed for the formation and precipitation of Cr_7_C_3_ carbide. The presence of larger particles in the molten pool inhibited grain growth, leading to compositional segregation and weakening of the directional growth of columnar grains.

The elemental distribution in the corresponding region of the Co coating is illustrated in Figure 10g–i. The surface microstructure exhibited a finer and more homogeneous morphology, predominantly consisting of columnar dendrites and interdendritic regions, devoid of discernible defects. Area 1 primarily comprises columnar dendritic structure, corroborated by XRD analysis, revealing the presence of Cr_7_C_3_ carbide and Cr_23_C_6_ carbide reinforcement phase. Meanwhile, Area 2, representing the interdendritic regions, exhibited a composition mainly characterized by Cr_23_C_6_ carbide and WO_3_. The precipitation of Cr_23_C_6_ is contingent upon the cooling rate of the coating, with higher thermodynamic driving forces facilitating its formation in the absence of WB particles hindrance.

The surface modification of the substrate is achieved through laser cladding of spherical tungsten boride (WB)-reinforced cobalt-based coatings, exhibiting no apparent pores or cracks. The reinforcement effect of the coating was primarily attributed to the addition of spherical WB particles. As the second phase, these particles dispersed within the γ-Co matrix, thereby enhancing crack resistance and providing structural support to the cobalt matrix, resulting in overall strengthening of the composite coating. Starting from the WB particles as nucleation sites, different microstructures were formed outward, including equiaxed grains, predominantly planar and cellular grains, surrounding the particles. Subsequently, columnar dendrites grew outward, solidified, and grew within the melting zone in the direction of the heat flow, ultimately exhibiting a well-arranged columnar dendritic microstructure.

**Figure 11:**
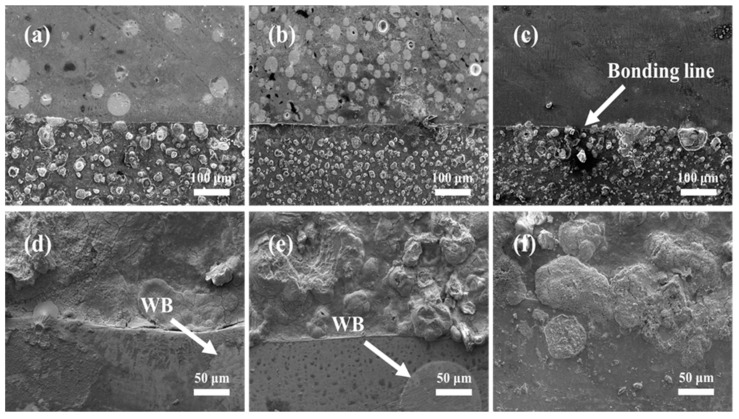
Cross-section SEM of laser cladded coatings: (**a**,**d**) Co + 15%WB; (**b**,**e**) Co + 45%WB; (**c**,**f**) Co.

Figure 11 shows the SEM image of the cross-section of the laser-cladded coating. As shown in Figure 11a–c, there is a bright and fine bonding line between the cladding layer and the substrate steel, indicating a good metallurgical bond between the coating and the substrate [27,28]. The spherical WB particles added to the coating exhibited high sphericity and uniform distribution, and the coating showed no obvious cracks or pores. In the Co + 45%WB coating, the spherical WB particles were uniformly dispersed and fused in the γ-Co matrix, resulting in effective dispersion strengthening. After etching, the spherical WB particles in the coating showed no significant signs of corrosion, while different degrees of corrosion morphologies were observed in the dendritic and interdendritic eutectic structures around the WB particles.

### 3.2. Hardness of the Laser Cladded Coatings

**Figure 12:**
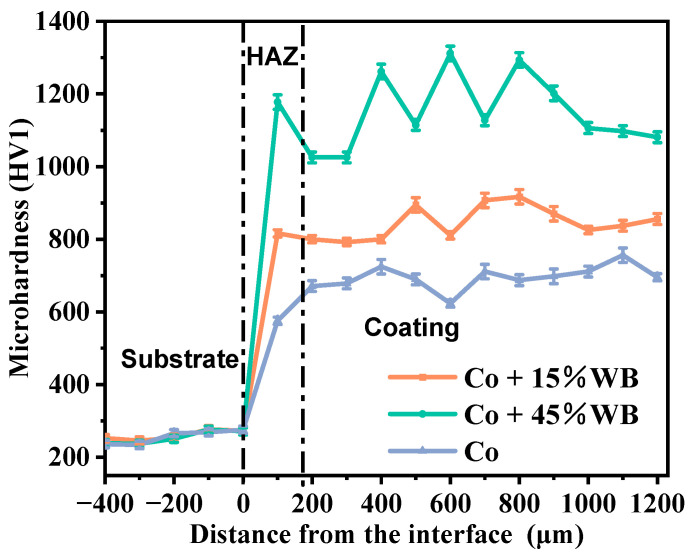
Cross-section microhardness distribution of the laser cladded coatings.

Microhardness information is an important indicator to analyze the mechanical properties of laser-cladded coatings [29,30]. To reveal the influence of spherical WB content on the hardness, the microhardness distribution of the coating in the depth direction is shown in Figure 12. The variation trend of microhardness of laser-cladding specimens can be divided into three distinct regions, namely the substrate zone, the heat affected zone (HAZ), and the cladded coating zone [31,32]. It can be seen that the microhardness of the cladded coating was significantly higher than that of the substrate, and the microhardness of the coating with the addition of spherical WB was also significantly higher than that of the Co-based coating. The average microhardness of Co + 15% WB, Co + 45% WB, and Co samples was, respectively, 3.28 times, 4.48 times, and 2.66 times to the substrate steel hardness (256.96 HV1). The average hardness of Co-based WB coating increased with the increase in WB content. When the coating contained 45% WB, the maximum hardness of the coating was 1152.01 HV1. The increase in hardness can be attributed to the strengthening effect of strengthening phases WB and Cr_7_C_3_ carbide. The microhardness of the Co + 45%WB coating fluctuates, indicating that more amorphous phases are generated in the Co + 45%WB coating, which has an impact on the hardness of the coating. The high hardness of Co-based WB coating is mainly due to the dispersion strengthening effect of WB on the coating. Meanwhile, the generation of carbides (Cr_7_C_3_ and Cr_23_C_6_) and γ-Co solid solution can significantly improve the microhardness of the coating and enhance the wear resistance of the coating. Previous studies have shown that the hardness of Co-based coatings with 30 wt.% WC addition is 411 HV_0.2_. When the addition amount is 60 wt.% WC, the hardness of Co-based WC coatings can reach as high as 701 HV_0.2_ [33]. The coating with spherical WB addition has higher hardness than the coating with WC addition, thus the development of Co-based WB coating has more application prospects.

**Figure 13:**
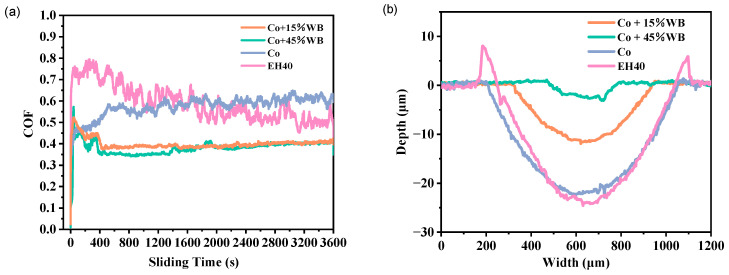
(**a**) Coefficient of friction of specimens in dry friction condition at −20 °C; (**b**) 2D profile of wear tracks in a dry friction at −20 °C.

The 2D wear track profiles of the three coatings and EH40 steel under dry sliding at −20 °C are presented in Figure 13. Table 4 summarizes the tribological properties of the specimens in dry sliding friction at −20 °C.

The wear rate (W) is calculated using the following formula [34]:

(1) W=V/(F×L)

where V (mm^3^) represents the wear volume, the total wear amount is the product of the track multiplied by the oscillation amplitude. F (N) represents the applied load, and L (m) represents the sliding distance. Under constant normal load and sliding distance, a larger wear volume corresponds to a higher wear rate.

It can be observed that EH40 exhibits the largest wear width and depth. The addition of spherical WB in Co + 15%WB and Co + 45%WB coatings enhances the hardness of the composite coatings by the formation of hardening phases such as Cr_23_C_6_ and Cr_2_Ni_3_ through rapid melting around WB particles. With increasing content of spherical WB, the hardness of the Co-based WB coatings gradually increases, resulting in reduced wear track depth and width, and significantly improved wear resistance. The relationship between hardness and wear volume follows Archard’s law [34]:

(2) $$K=\frac{V}{F_{N}\times L}H$$

where V is the wear volume of the specimen, FN is the applied load, L is the sliding distance, and H is the microhardness of the coating. Archard’s law states that the wear volume is inversely proportional to the material hardness [34].

The wear volume of EH40 is 4.600 mm^3^, indicating the highest wear volume. By comparing the wear volumes of Co + 15%WB, Co + 45%WB, and Co, it is observed that the wear volume gradually decreases with the addition of WB. Co + 45%WB exhibits the lowest wear volume, measuring 0.128 mm^3^.

### 3.3. Low-Temperature Wear Resistance

Figure 13a shows the coefficient of friction (COF) curves of three coatings (Co, Co + 15%WB, and Co + 45%WB) and the substrate EH40 steel in dry friction condition at −20 °C. The dynamic coefficients of the tested specimens are related to the state of the friction process. The wear resistance mainly depends on their hardness, surface microstructure, deformability, and crack propagation resistance [35]. During reciprocating friction and ball-on-disc wear processes, two wear stages can be distinguished: initial wear and steady-state wear. As shown in Figure 13a, during the initial running-in friction stage, the WC balls come into contact with the sample surface under the preset load, leading to a rapid increase in the friction coefficient between the coatings and the substrate steel. This may be attributed to the sudden increase in the contact area between the sample surface and the ball when subjected to compressive stress load, resulting in an increase in friction force with the contact area enlargement and plastic deformation on the sample surface [36]. As the friction process continues, the normal compressive stress gradually transforms into horizontal shear forces, and the friction enters the steady-state stage, where the fluctuations in the curve reduce to some extent. In particular, the Co + 15%WB and Co + 45%WB coatings exhibit a decreasing trend and tend to stabilize. This is because the spherical WB particles play a supportive role in the Co-based coatings. When encountering compressive stress, the spherical particles immerse into the alloy, effectively dispersing the stress. On the other hand, the coefficient fluctuations of the Co coating and EH40 are unstable. The friction between the friction surface and the ball causes the detachment of the hard phases and some metal coating, leading to changes in the friction state of the surface and affecting the fluctuations of the COF.

The average COF of specimens under dry sliding friction at −20 °C is shown in Table 4. EH40 exhibited the highest friction coefficient (0.5918), slightly higher than the Co coating (0.5743). EH40 demonstrated significant fluctuations during the friction process, as the ferrite and pearlite phases in EH40 exhibited significant differences in wear resistance, resulting in the generation of numerous of wear debris. The COF of Co + 15%WB (0.4003) and Co + 45%WB (0.3807) were lower than those of EH40 and the Co coating. The higher the content of spherical WB particles added, the better the wear resistance of the coatings. The addition of WB particles helps prevent plastic deformation and plowing effects caused by the compression between the friction ball and the coating surface.

**Figure 14:**
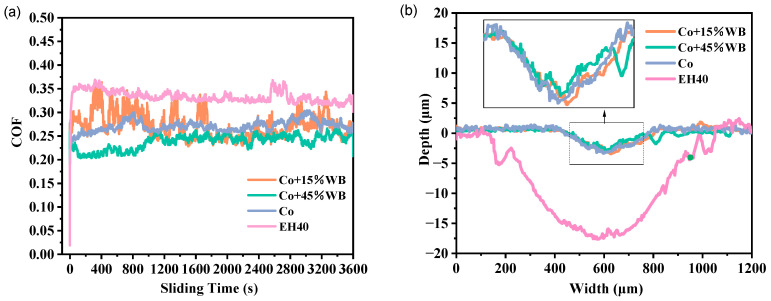
(**a**) Coefficient of friction of specimens in 3.5 wt.% NaCl solution at -20℃, (**b**) 2D profile of wear tracks in 3.5 wt.% NaCl solution at −20 °C (The inserted image is the magnified 2D profile of Co, Co + 15%WB, and Co + 45%WB coatings).

Figure 14a shows the COF curves of the three coatings and the EH40 substrate in the 3.5 wt.% NaCl solution at −20 °C. The fluctuation of the friction coefficient is influenced by the lubricating medium during the friction process. Unlike dry friction, in the friction pair with a fluid medium, a hybrid friction consisting of fluid friction and interfacial friction is formed due to the lubrication effect of fluid dynamics. This widens the friction regime and facilitates the formation of a reaction film to reduce friction and wear. The friction coefficient in wet friction is significantly lower than that in dry friction. During the friction process, a thin liquid film adheres to the surface of the abrasive ball, dispersing the load. The friction coefficients of the Co, Co + 15%WB, and Co + 45%WB coatings are similar and significantly lower than that of EH40 (0.3334). Among them, the Co + 45%WB coating exhibits the lowest friction coefficient (0.2384). This demonstrates that Co + 45%WB coating possess excellent wear resistance in the 3.5 wt.% NaCl solution at −20 °C.

Figure 14b illustrates the 2D profile of the three coatings and EH40 steel wear tracks in 3.5 wt.% NaCl solution at −20 °C. It can be observed that EH40 exhibits the largest width and depth of wear track in the 3.5 wt.% NaCl solution. Ferrite has a higher adhesive coefficient than pearlite, but compared to the Co-based alloy, metals with higher atomic density and lower surface energy exhibit lower adhesive properties. As a result, EH40 has poor wear resistance. In low-temperature friction, seawater freezes and fractures under the compression of the abrasive ball, forming crushed ice. This hinders direct contact between the coating and the abrasive ball interface, leading to a decrease in COF and wear rate. In the presence of lubrication, the dynamic load is transmitted through the liquid film, which is prone to fatigue wear under compressive stress. Table 5 lists the tribological properties of the specimens in 3.5 wt.% NaCl solution at −20 °C. Higher coating hardness, elastic modulus, and strength enhance the ability to withstand fluid impact and load transfer. The data of 2D frictional scratches of the coatings align with the magnitude of the friction coefficient. The lower the friction coefficient, the shallower the wear track. Therefore, the Co + 45%WB coating exhibits the smallest width and depth of frictional wear.

Co + 15%WB coating exhibits slightly higher wear depth compared to the Co coating. This may be attributed to abrasive erosion occurring during the friction process. During reciprocating friction, corrosion products and spherical WB particles are dislodged, creating relative motion parallel to the wear surface. The particles undergo downward impact due to centrifugal force, resulting in wave-like depressions at the bottom of the wear track.

### 3.4. Tribological Mechanism under Low Temperature

**Figure 15:**
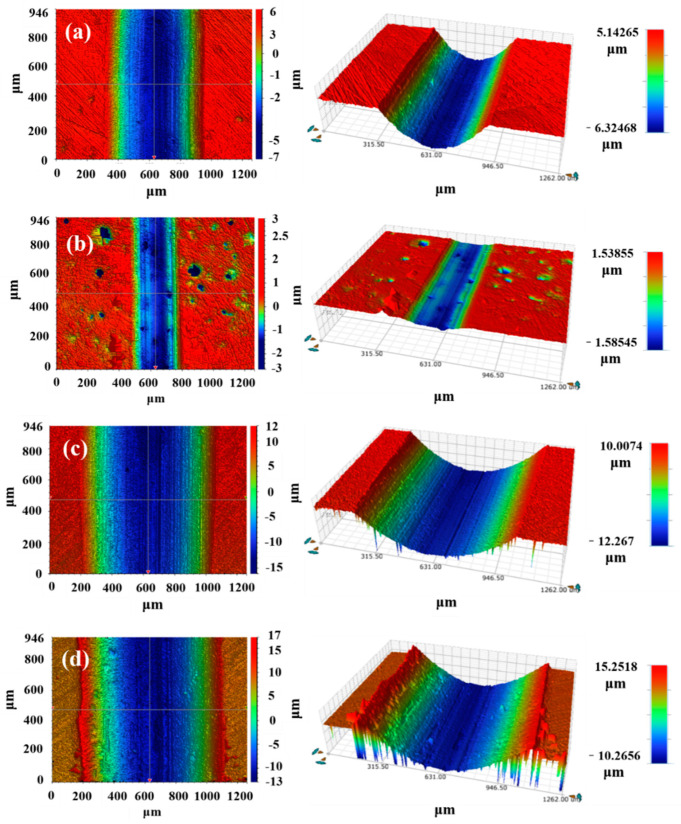
3D profile of the wear tracks under dry sliding at −20 °C: (**a**) Co + 15%WB; (**b**) Co + 45%WB; (**c**) Co, (**d**) EH40.

Figure 15 shows the 3D wear morphology of three coatings and EH40 steel under dry sliding at −20 °C. The wear depth is represented by different colors in the white light interferograms. Red indicates the region with no wear, green represents moderate wear, and blue represents severe wear. The Co + 45%WB coating exhibits the smallest wear width and depth, indicating the lowest degree of wear. The surface of the coating still contains numerous WB particles, which effectively hinder sliding friction and reduce wear. The surface of the Co coating is smoother compared to the WB-added Co+15%WB and Co + 45%WB coatings. Since there is no WB present in the Co coating, the wear volume is significantly higher. In the blue scratch region, fine scratches are generated due to the abrasive wear of hard phases such as Cr_23_C_6_ and Cr_7_C_3_ in the Co coating during friction. Under the compression of WC abrasive balls, the wear debris particles repeatedly make dense contact with the surface, forming plowing scratches on the surface of the Co coating.

**Figure 16:**
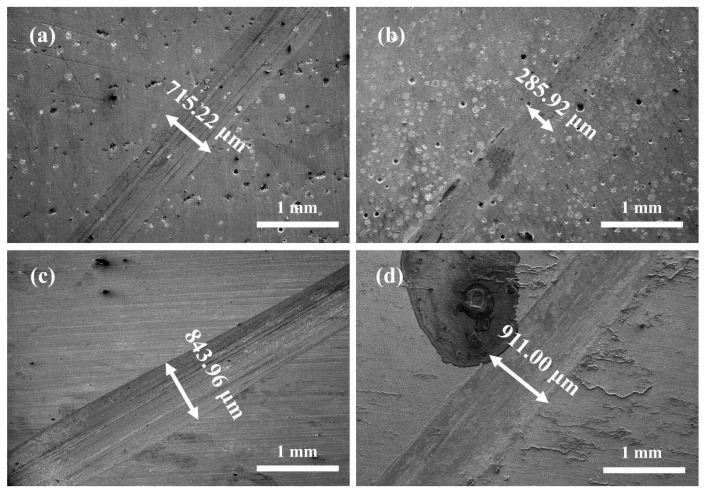
SEM images of surface wear track under dry sliding friction at −20 °C: (**a**) Co + 15%WB; (**b**) Co + 45%WB; (**c**) Co, (**d**) EH40.

Figure 16 shows the SEM images of the wear surface of three coatings and EH40 steel under dry sliding at −20 °C. From the wear tracks of the Co + 15%WB and Co + 45%WB coatings (Figure 16a,b), evident signs of abrasive wear are observed on the rough surface of the WB-added coatings, and the surface depressions may be attributed to the detachment of hard phases under lateral stress. When sliding occurs around the WB particles, during the wear process between the coating surface and the opposing abrasive ball, the hard particles can effectively resist the normal and tangential loads, but the softer surface layers surrounding the particles are prone to deformation. In the steady-state friction stage, the load-bearing surfaces on the wear track undergo cyclic loading due to static friction. The wear debris undergoes plastic shear deformation and accumulates under repeated tangential loading.

**Figure 17:**
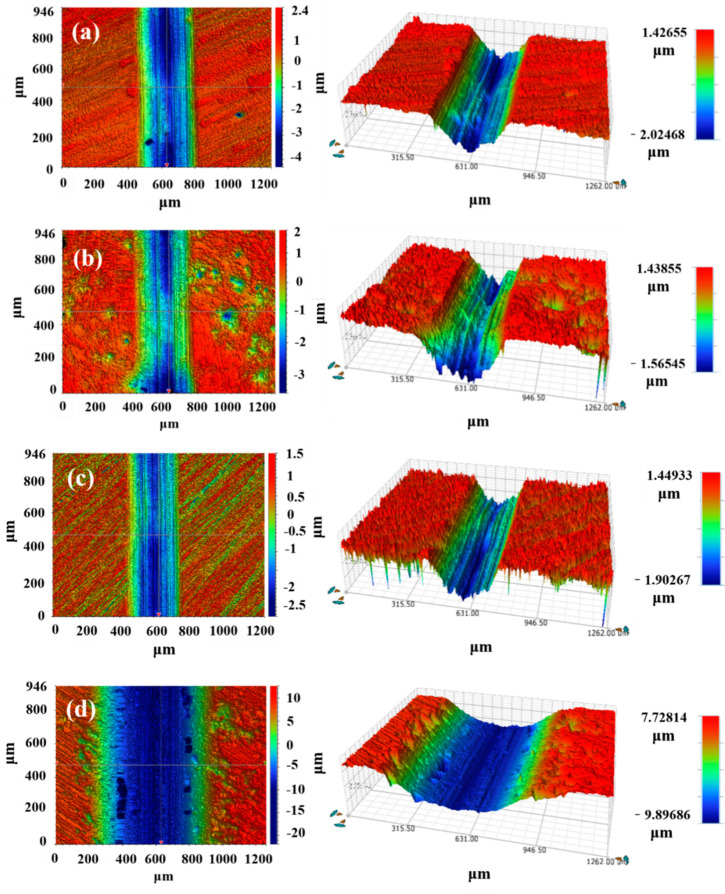
3D profile of wear tracks in 3.5 wt.% NaCl solution at −20 °C: (**a**) Co + 15%WB; (**b**) Co + 45%WB; (**c**) Co; (**d**) EH40.

Figure 17 shows the 3D wear morphology of the three coatings and EH40 steel in the 3.5 wt.% NaCl solution at −20 °C. It can be observed that the small red protrusions in the Co + 15%WB and Co + 45%WB coatings are spherical WB particles, indicating a relatively rough surface. The blue regions at the bottom of the wear tracks reveal pronounced ploughing morphology. This is mainly due to the entrainment of hard phase abrasives by the fluid, resulting in fine scratches under the compression of the wear ball. In terms of wear volume, Co + 45%WB (0.051) < Co (0.057) < Co + 15%WB (0.067), indicating that the detachment of particles and the influence of hard phases on the friction process in the 3.5 wt.% NaCl solution are significant. The addition of spherical WB particles improves the wear resistance of the coatings in the 3.5 wt.% NaCl solution. Due to the plastic deformation of micro-convexes in the fluid-friction pair, the coefficient of friction is inversely proportional to the pressure transmitted by the fluid [37]. The more wear-resistant the coating is, the better its ability to resist the transfer of dynamic loads by the lubrication fluid. Due to its relatively smooth surface, the Co coating experiences less wear from large-sized abrasive particles under the same fluid load, resulting in narrower wear tracks compared to Co + 15%WB and Co + 45%WB coatings.

**Figure 18:**
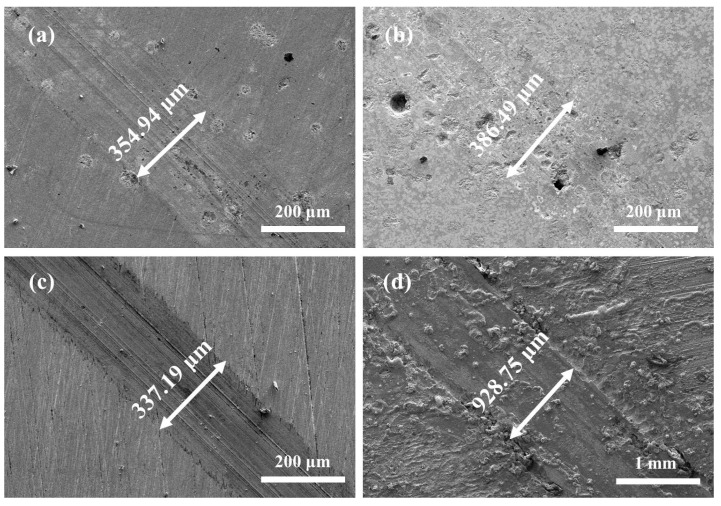
SEM profile of wear tracks in 3.5 wt.% NaCl solution at −20 °C: (**a**) Co + 15%WB; (**b**) Co + 45%WB; (**c**) Co; (**d**) EH40.

Figure 18 presents the SEM images of surface wear track in 3.5 wt.% NaCl solution at −20 °C. Due to the lubrication of 3.5 wt.% NaCl solution, the tracks on the surfaces of the three coatings are relatively shallow, exhibiting distinct elongated plough grooves. As shown in Figure 18a,b, there are evident voids resulting from the detachment of spherical WB particles within the scratches. The wear mechanisms of Co + 15%WB and Co + 45%WB coatings involve abrasive wear and abrasive particle erosion. The Co coating surface also shows noticeable scratches and some debris. The wear mechanism of the Co coating is attributed to abrasive particle wear. EH40 steel exhibits the poorest frictional performance, characterized by the largest depth and width of surface scratches, along with a considerable amount of residual wear debris. The wear mechanism of EH40 steel primarily involves fatigue wear.

**Figure 19:**
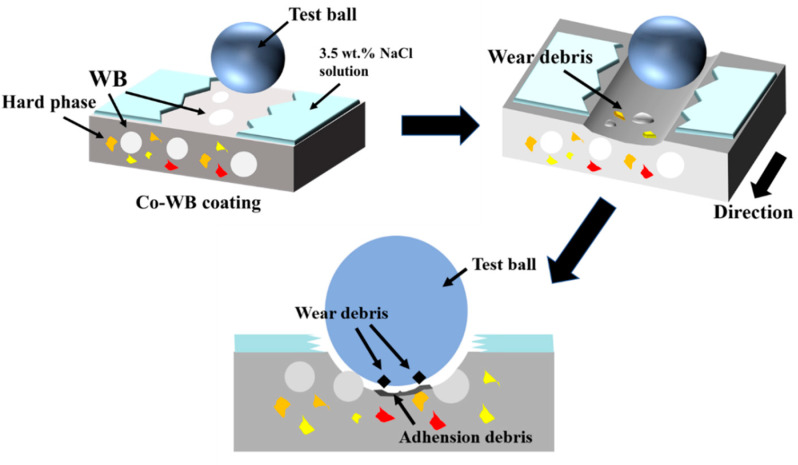
Schematic diagram of friction process of Co-WB coatings under low temperature.

Figure 19 illustrates the schematic diagram of the wear process of Co-WB coatings. When the WC ball contacts the coating surface, compressive stress is exerted on the coating surface, causing the softer regions around the spherical WB particles to undergo plastic deformation. This results in the generation of a large number of wear debris through adhesive and ploughing actions. Some of the debris undergoes adhesive wear due to the repeated transverse loading of the WC ball, while others are expelled from the wear scar by the action of the ball. In the presence of the 3.5 wt.% NaCl solution, a significant amount of wear debris dissolves in the solution, leading to abrasive particle wear. In the stable friction stage, boundary friction occurs between the WC ball and the spherical WB particles, which are two different hard ceramics. Some of the spherical WB particles fractured under the influence of transverse loading, while others resisted the cutting forces of the WC ball within the Co-based alloy. The motion of the frictional debris causes abrasive particle wear. In the presence of the 3.5 wt.% NaCl solution, the frictional debris is entrapped within the liquid medium and undergoes abrasive particle wear under the dynamic loading of the WC through the liquid film.

## 4. Conclusions

In summary, three groups of Co-based coatings reinforced with different spherical WB particles were successfully prepared on the EH40 steel by laser-cladded technology, and a good metallurgical bonding with the substrate steel was obtained. Cr_7_C_3_ and Cr_23_C_6_ carbides played an important role in strengthening the cladding layers. Around the WB particles, equiaxed grains were formed, and as dendrites grew outward, columnar dendrites were formed. The hardness of the cladded layers increased with an increase in the content of spherical WB, primarily due to the dispersion strengthening effect of WB on the coatings.

The addition of more spherical WB resulted in lower friction coefficients under dry sliding friction conditions at −20 °C. Both the Co + 15%WB and Co + 45%WB coatings experienced particle detachment of WB during the friction process, and the main wear mechanism was abrasive wear. The Co coating exhibited scratches caused by Cr_7_C_3_ carbides on the wear tracks, and the entrapped wear debris experienced adhesive wear and abrasive wear.

Compared with dry sliding, due to the plastic deformation of micro-convexes and lubrication function in the 3.5 wt.% NaCl solution, the wear tracks on the surfaces of the three tested coatings were shallower, exhibiting distinct elongated plough grooves. The presence of WB detachment holes in both the Co + 15%WB and Co + 45%WB coatings indicates that the primary wear mechanisms are abrasive wear and abrasive particle erosion.

## Figures and Tables

**Table 1:** Process parameters of spheroidized WB.

Parameters	Set Value
Feed rate (g/min)	20
Carrier gas (L/min)	5
Dispersing gas (L/min)	10
Central gas (L/min)	30
Sheath gas(L/min)	30
Pressure (kPa)	15
Power (kW)	55

**Table 2:** Mixed ratio of the composite powders.

Samples	WB Composition (wt.%)	Co-Based Powder (wt.%)
Co + 15%WB	15	85
Co + 45%WB	45	55
Co	0	100

**Table 3:** Laser cladded parameters.

Laser Powder (kW)	Laser Wavelength (nm)	Scanning Speed (mm/s)	Powder Feed Rate (g/min)	Spot Diameter (mm)	Overlapping Ratio
5.5	1080	16	33	5	33%

**Table 4:** Tribological properties of the specimens in dry sliding friction at −20 °C.

Specimens	Wear Width (μm)	Wear Depth (μm)	Wear Volume (mm^3^)	Wear Rate (mm^3^/N·m^−1^)	Average COF
Co + 15%WB	715.223	11.195	0.984	2.733 × 10^−3^	0.4003
Co + 45%WB	285.921	3.124	0.128	3.567 × 10^−4^	0.3807
Co	843.960	22.435	3.278	9.105 × 10^−3^	0.5743
EH40	911.003	24.208	4.600	12.78 × 10^−3^	0.5918

**Table 5:** Tribological properties of the specimens in 3.5 wt.% NaCl solution at −20 °C.

Specimens	Wear Width (μm)	Wear Depth (μm)	Wear Volume (mm^3^)	Wear Rate (mm^3^/N·m^−1^)	Average COF
Co + 15%WB	354.936	3.440	0.067	0.186 × 10^−4^	0.2729
Co + 45%WB	386.486	2.902	0.051	0.142 × 10^−4^	0.2384
Co	337.189	3.352	0.057	0.158 × 10^−4^	0.2709
EH40	928.749	17.615	0.778	1.080 × 10^−4^	0.3334

## Data Availability

The data generated and analyzed during the current study are not publicly available due to data that may be used in the next research, but are available from the corresponding author on reasonable request.
